# Supplementary material for: HIVprotI: an integrated web based platform for prediction and design of HIV proteins inhibitors
Source: J Cheminform. 2018 Mar 9;10:12. doi: 10.1186/s13321-018-0266-y (PMC5845081; doi:10.1186/s13321-018-0266-y)
Supplement: Supplementary file 2 — Additional file 2. Source code of HIVProtI web server. [file 13321_2018_266_MOESM2_ESM.zip › HIVprotI_Source-code/hivprotI/weka-3-6-6/doc/weka/classifiers/evaluation/NominalPrediction.html]

NominalPrediction


---


|  |  |  |
| --- | --- | --- |
| **Overview** \| **Package** \| **Class** \| **Tree** \| **Deprecated** \| **Index** \| **Help** \| **Weka's home** | |  |
| **PREV CLASS**   **NEXT CLASS** | **FRAMES**    **NO FRAMES**     **All Classes** |  |
| SUMMARY: NESTED \| FIELD \| CONSTR \| METHOD | DETAIL: FIELD \| CONSTR \| METHOD |  |


---


## weka.classifiers.evaluation Class NominalPrediction

```
java.lang.Object
  weka.classifiers.evaluation.NominalPrediction
```

**All Implemented Interfaces:**: java.io.Serializable, Prediction, RevisionHandler

---

``` public class NominalPrediction extends java.lang.Object implements Prediction, java.io.Serializable, RevisionHandler ```

Encapsulates an evaluatable nominal prediction: the predicted probability
distribution plus the actual class value.

**Version:**
:   $Revision: 1.12 $

**Author:**
:   Len Trigg (len@reeltwo.com)

**See Also:**: Serialized Form

---

**Field Summary**

**Fields inherited from interface weka.classifiers.evaluation.Prediction**

`MISSING_VALUE`


**Constructor Summary**

`NominalPrediction(double actual,
double[] distribution)`
  
          Creates the NominalPrediction object with a default weight of 1.0.

`NominalPrediction(double actual,
double[] distribution,
double weight)`
  
          Creates the NominalPrediction object.


| **Method Summary** | |
| --- | --- |
| `double` | `actual()`             Gets the actual class value. |
| `double[]` | `distribution()`             Gets the predicted probabilities |
| `java.lang.String` | `getRevision()`             Returns the revision string. |
| `static double[]` | `makeDistribution(double predictedClass, int numClasses)`             Convert a single prediction into a probability distribution with all zero probabilities except the predicted value which has probability 1.0. |
| `static double[]` | `makeUniformDistribution(int numClasses)`             Creates a uniform probability distribution -- where each of the possible classes is assigned equal probability. |
| `double` | `margin()`             Calculates the prediction margin. |
| `double` | `predicted()`             Gets the predicted class value. |
| `java.lang.String` | `toString()`             Gets a human readable representation of this prediction. |
| `double` | `weight()`             Gets the weight assigned to this prediction. |

**Methods inherited from class java.lang.Object**

`equals, getClass, hashCode, notify, notifyAll, wait, wait, wait`

**Constructor Detail**

### NominalPrediction

```
public NominalPrediction(double actual,
                         double[] distribution)
```

:   Creates the NominalPrediction object with a default weight of 1.0.

    **Parameters:**: `actual` - the actual value, or MISSING\_VALUE.: `distribution` - the predicted probability distribution. Use NominalPrediction.makeDistribution() if you only know the predicted value.

---


### NominalPrediction

```
public NominalPrediction(double actual,
                         double[] distribution,
                         double weight)
```

:   Creates the NominalPrediction object.

    **Parameters:**: `actual` - the actual value, or MISSING\_VALUE.: `distribution` - the predicted probability distribution. Use NominalPrediction.makeDistribution() if you only know the predicted value.: `weight` - the weight assigned to the prediction.


**Method Detail**

### distribution

```
public double[] distribution()
```

:   Gets the predicted probabilities

    :   **Returns:**: the predicted probabilities

---


### actual

```
public double actual()
```

:   Gets the actual class value.

    :   **Specified by:**: `actual` in interface `Prediction`
    :   **Returns:**: the actual class value, or MISSING\_VALUE if no prediction was made.

---


### predicted

```
public double predicted()
```

:   Gets the predicted class value.

    :   **Specified by:**: `predicted` in interface `Prediction`
    :   **Returns:**: the predicted class value, or MISSING\_VALUE if no prediction was made.

---


### weight

```
public double weight()
```

:   Gets the weight assigned to this prediction. This is typically the weight
    of the test instance the prediction was made for.

    :   **Specified by:**: `weight` in interface `Prediction`
    :   **Returns:**: the weight assigned to this prediction.

---


### margin

```
public double margin()
```

:   Calculates the prediction margin. This is defined as the difference
    between the probability predicted for the actual class and the highest
    predicted probability of the other classes.

    :   **Returns:**: the margin for this prediction, or MISSING\_VALUE if either the actual or predicted value is missing.

---


### makeDistribution

```
public static double[] makeDistribution(double predictedClass,
                                        int numClasses)
```

:   Convert a single prediction into a probability distribution
    with all zero probabilities except the predicted value which
    has probability 1.0. If no prediction was made, all probabilities
    are zero.

    :   **Parameters:**: `predictedClass` - the index of the predicted class, or MISSING\_VALUE if no prediction was made.: `numClasses` - the number of possible classes for this nominal prediction. **Returns:**: the probability distribution.

---


### makeUniformDistribution

```
public static double[] makeUniformDistribution(int numClasses)
```

:   Creates a uniform probability distribution -- where each of the
    possible classes is assigned equal probability.

    :   **Parameters:**: `numClasses` - the number of possible classes for this nominal prediction. **Returns:**: the probability distribution.

---


### toString

```
public java.lang.String toString()
```

:   Gets a human readable representation of this prediction.

    :   **Overrides:**: `toString` in class `java.lang.Object`
    :   **Returns:**: a human readable representation of this prediction.

---


### getRevision

```
public java.lang.String getRevision()
```

:   Returns the revision string.

    :   **Specified by:**: `getRevision` in interface `RevisionHandler`
    :   **Returns:**: the revision


---


|  |  |  |
| --- | --- | --- |
| **Overview** \| **Package** \| **Class** \| **Tree** \| **Deprecated** \| **Index** \| **Help** \| **Weka's home** | |  |
| **PREV CLASS**   **NEXT CLASS** | **FRAMES**    **NO FRAMES**     **All Classes** |  |
| SUMMARY: NESTED \| FIELD \| CONSTR \| METHOD | DETAIL: FIELD \| CONSTR \| METHOD |  |


---
